# Supplementary material for: Structural and Functional Analysis of the Human IQSEC2 S1474Qfs*133 Mutation
Source: Biomolecules. 2025 Apr 29;15(5):635. doi: 10.3390/biom15050635 (PMC12109076; doi:10.3390/biom15050635)
Supplement: Supplementary file 1 [file biomolecules-15-00635-s001.zip › biomolecules-3594988-supplementary.pdf]

Supplementary Figures and Tables

Supplementary Figures S1-S9

Figure S1. Amino acid sequence of human IQSEC2 S1474Qfs*133

MEAGSGPPGGPGSESPNRAVEYLLELNNIIESQQQLLETQRRRIEELEGQLDQLTQENRDLREESQLHRGELHRDPHGA
RDSPGRESQYQNLRETQFHHRELRESQFHQAARDVGYPNREGAYQNREAVYRDKERDASYPLQDTTGYTARERDVAQ
CHLHHENPALGRERGGREAGPAHPGREKEAGYSAAVGVGPRPPRERGQLSRGASRSSSPGAGGGHSTSTSTSPATTLQ
RKSDGENSRTVSVEGDAPGSDLSTAVDSPGSQPPYRLSQLPPSSSHMGGPPAGVGLPWAQRARLQPASVALRKQEEE
EIKRSKALSDSYELSTDLQDKKVEMLERKYGGSFLSRRAARTIQTAFRQYRMNKNFERLRSSASESRMSRRIILSNMRMQ
FSFEEYEKAQNPAYFEGKPASLDEGAMAGARSHRLERGLPYGGSCGGGIDGGGSSVTTSGEFSNDITELEDSFSKQVKSL
AESIDEALNCHPSGPMSEEPGSAQLEKRESKEQQEDSSATSFSDLPLYLDDTVPQQSPERLPSTEPPPQGRPEFWAPAPL
PPVPPPVPSGTREDGSREEGTRRGPGCLECRDFRLRAAHLPLLTIEPPSDSSVDLSDRSDRGSVHRQLVYEADGCSPHGT
LKHKGPPGRAPIPHRHYPAPEGPAPAPPGPLPPAPNSGTGPSGVAGGRRLGKCEAAGENSDGGDNESLESSSNSNETI
NCSSGSSSRDSLREPPATGLCKQTYQRETRHSWDSPAFNNDVVQRRHYRIGLNLFNKKPEKGIQYLIERGFLSDTPVGVA
HFILERKGLSRQMIGEFLGNRQKQFNRDVLDCVVDEMDFSSMDLDDALRKFQSHIRVQGEAQKVERLIEAFSQRYCVC
NPALVRQFRNPDTIFILAFAIILLNTDMYSPSVKAERKMKLDDFIKNLRGVDNGEDIPRDLLVGIYQRIQGRELRTNDDHV
SQVQAVERMIVGKKPVLSLPHRRLVCCCQLYEVPDPNRPQRLGLHQREVFLFNDLLVVTKIFQKKKILVTYSFRQSFPLVE
MHMQLFQNSYYQFGIKLLSAVPGGERKVLIIFNAPSLQDRLRFTSDLRESIAEVQEMEKYRVESELEKQKGMMRPNAS
QPGGAKDSVNGTMARSSLEDTYGAGDGLKRGALSSSLRDLSDAGKRGRRNSVGSLDSTIEGSVISSPRPHQRMPPPPP
PPPPEEYKSQRPVSNSSSFLGSLFGSKRGKGPFQMPPPPTGQASASSSSASSTHHHHHHHHHGHSHGGLGVLPDGQSK
LQALHAQYCQGPGPAPPPYLPPQQPSLPPPPQQPPPLPQLGSIPPPPASAPPVGPHRHFHAHGPVPGPQHYTLGRPGR
APRRGAGGHPQFAPHGRHPLHQPTSPLPLYSPAPQHPPAHKQGPKHFIFSHHPQMMPAAGAAGGPGSRPPGGSYS
HPHHPQSPLSPHSPIPPHPSYPPLPPPSPHTPHSPLPPTSPHGPLHASGPPGTANPPQCKPQGQAKPDQHRGLMNGES
ELGNRKVWGATGEGPCTLFASSVFSKIYIHIAMSSSPCPPCLGTRPLTPSSCHGVSFQIRGSLPLEAWTQGAPALGSGEG
LPEILVLFLPSSSSPLFPGGLALWPQTSGPV

Figure S2. Targetting construct

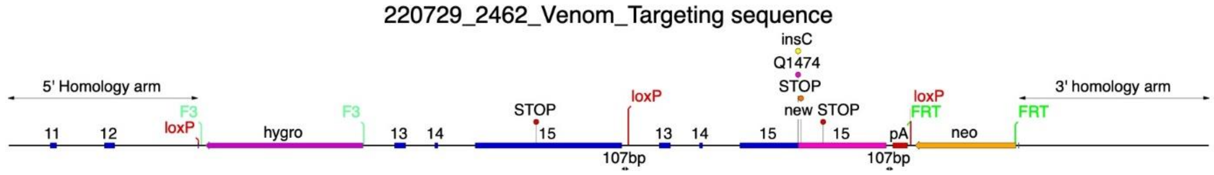

Figure S3. Location of homology arms

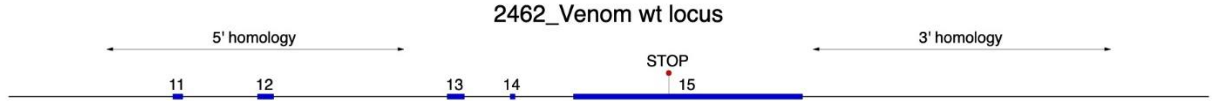

Figure S4. Targetted locus

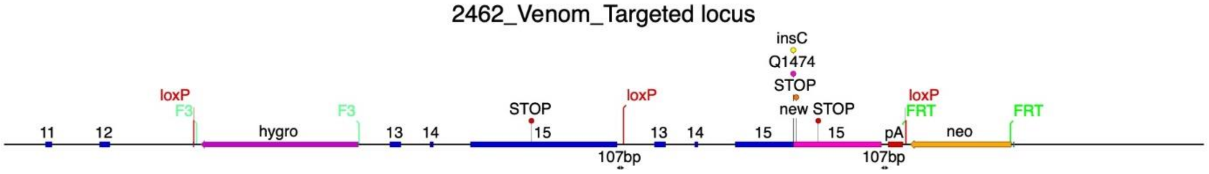

Figure S5. Map of conditional allele.

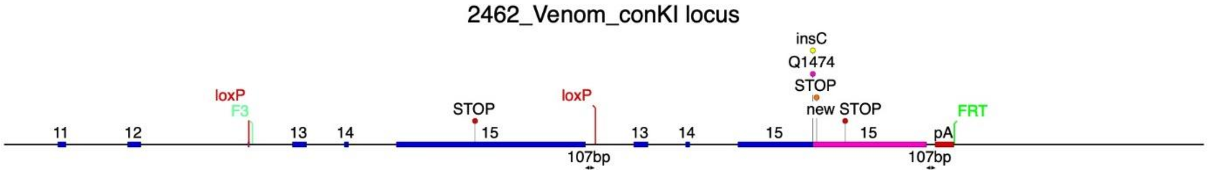

Figure S6. Map of knock in allele.

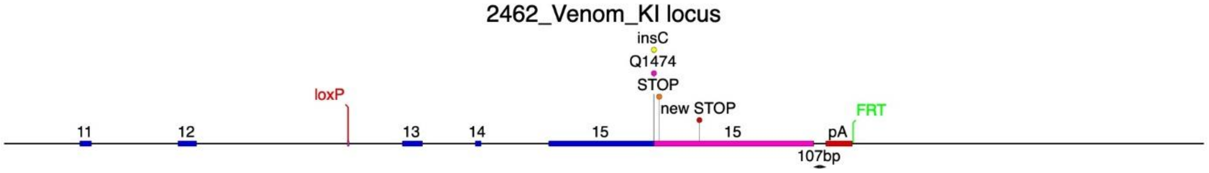

Figure S7. Amino acid sequence of humanized mouse IQSEC2 S1474Qfs*133 protein.

MEAGSGPPGGPGSESPNRAVEYLLELNNIIESQQQLLETQRRRIEELEGQLDQLTQENRDLREESQLHRGELHRDPLGA
RDSPGRESQYQNLRETQFHHRELRESQFHQASRDVGYPNRDGAYQNREAIYRDKEREASYQLQDTTGYTARERDVAQ
CHLHHENPALGRERGGREAGPAHPGREKEAGYSAAVGVGQRPPRERGQLSRGASRSSSPGAGGGHSTSTSTSPATTL
QRKSDGENSRTVSVEGDAPGSDLSTAVDSPGSQPPYRLSQLPPTSSHMGGPPAGVGLPWAQRARLQPASVALRKQEE
EEIKRSKALSDSYELSTDLQDKKVEMLERKYGGSFLSRRAARTIQTAFRQYRMNKNFERLRSSASESRMSRRIILSNMRM
QFSFEEYEKAQNPAYFEGKPASLDEGAMAGARSHRLERGLPYGGSCGGGIDGGGSSVTTSGEFSNDITELEDSFSKQVK
SLAESIDEALNCHPSGPMSEEPGSAQLEKRESKEQQEDSSATSFSDLPLYLDDPVPPPSPERLPSTEPPPQGRPEFWAPA
PLPPVPPPMPPGTREDGSREEGTRRGPGCLECRDFRLRAAHLPLLTIEPPSDSSVDLSDRSDRGSVHRQLVYEADGCSPH
GTLKHKGPPGRAPIPHRHYPAPEGPAPAPPGPLPPAPNSGTGPSGVAGGRRLGKCEAAGENSDGGDNESLESSSNSNE
TINCSSGSSSRDSLREPPATGLCKQTYQRETRHSWDSPAFNNDVVQRRHYRIGLNLFNKKPEKGIQYLIERGFLSDTPVG
VAHFILERKGLSRQMIGEFLGNRQKQFNRDVLDCVVDEMDFSSMDLDDALRKFQSHIRVQGEAQKVERLIEAFSQRYC
VCNPALVRQFRNPDTIFILAFAIILLNTDMYSPSVKAERKMKLDDFIKNLRGVDNGEDIPRDLLVGIYQRIQGRELRTNDD
HVSQVQAVERMIVGKKPVLSLPHRRLVCCCQLYEVPDPNRPQRLGLHQREVFLFNDLLVVTKIFQKKKILVTYSFRQSFP
LVEMHMQLFQNSYYQFGIKLLSAVPGGERKVLIIFNAPSLQDRLRFTSDLRESIAEVQEMEKYRVESELEKQKGMMRPN
ASQPGGAKDSVNGTLARSSLEDTYGAGDGLKRGALSSSLRDLSDAGKRGRRNSVGSLDSTIEGSVISSPRPHQRMPPPP
PPPPPEEYKSQRPVSNSSSFLGSLFGSKRGKGPFQMPPPPTGQASASSSSASSTHHHHHHHHHGHSHGGLGVLPDGQS
KLQALHAQYCQGPGPAPPPYLPPQQPPLPPPPQQPPPLPQLGSIPPPPASAPPVGPHRHFHAHGPVPGPQHYTLGRP
GRAPRRGAGGHPQFAPHGRHPLHQPTSPLPLYSPAPQHPPAHKQGPKHFIFSHHPQMMPAAGAAGGPGSRPPGGS
YSHPHHPQSPLSPHSPIPPHPSYPPLPPPSPHTPHSPLPPTSPHGPLHASGPPGTANPPQCKPQGQAKPDQHRGLMNG
ESELGNRKVWGATGEGPCTLFASSVFSKIYIHIAMSSSPCPPCLGTRPLTPSSCHGVSFQIRGSLPLEAWTQGAPALGSGE
GLPEILVLFLPSSSSPLFPGGLALWPQTSGPV

Figure S8. Schematic summary of breeding scheme to generate conKI and KI mice.

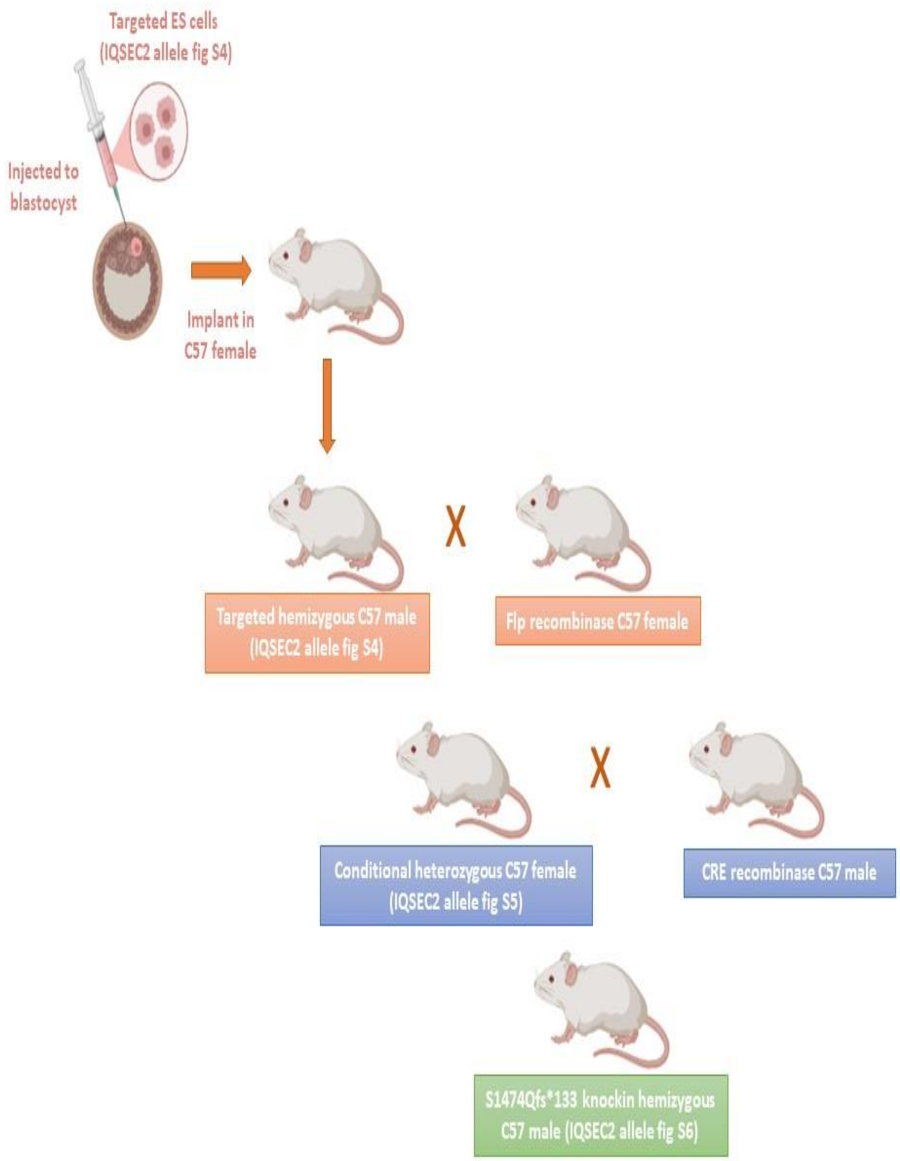

Figure S9. Western blot of brain IQSEC2 protein in wild type and S1474Qfs*133 mice. Western blot was performed as described in methods. Blot shows results for two wild type (WT mouse #1 and WT mouse #3) and two S1474Qfs*133 mice (Mut mouse #2 and Mut mouse #4) with samples run in duplicate for each mouse except for mouse 4. The IQSEC2 protein band is indicated with an arrow.

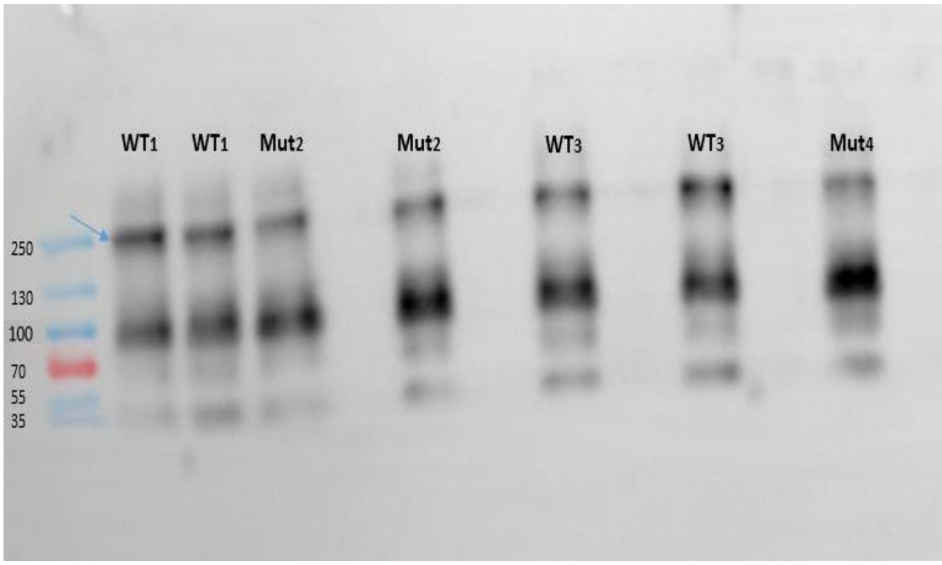

Figure S10. Quantitation of IQSEC2 mRNA in wild type and S1474Qfs*133 male mice brains.

qRT-PCR was performed as described in methods. Results are presented for 6 wild type (wt) and 5 S1474Q*fs133 (mut) male mice. There was no significant difference in the amount of IQSEC2 mRNA between the wt and mut mice: mean +/-SD [1.22+/-0.90 for wt vs 1.21+/-0.48 for mut; p=0.99].

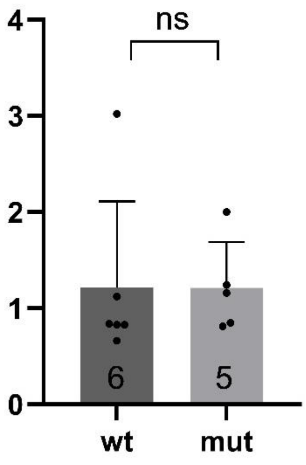

Supplementary Tables S1-S3

Table S1. Raw data of vocalizations in MT and WT mice

Name	USV		Noise	USV	
File name	Cluster_1	Cluster_2	Cluster_3	Cluster_4	**Sum_of_all_clusters_without_noise**
WT_9218.mat	46	4	1	0	**50**
WT_9220.mat	366	12	37	5	**383**
WT_9224.mat	613	0	1	2	**615**
WT_9225.mat	2129	29	9	30	**2188**
WT_9227.mat	37	0	0	0	**37**
WT_9228.mat	184	29	0	0	**213**
WT_9239.mat	142	0	0	13	**155**
WT_9241.mat	27	0	0	5	**32**
WT_9242.mat	41	0	1	0	**41**
MutS1474Q_9219.mat	0	0	2	0	**0**
MutS1474Q_9221.mat	0	0	0	0	**0**
MutS1474Q_9222.mat	0	0	0	0	**0**
MutS1474Q_9223.mat	0	0	0	0	**0**
MutS1474Q_9226.mat	0	0	0	0	**0**
MutS1474Q_9240.mat	0	0	0	0	**0**

Table S2. Primer design for quantitating IQSEC2 mRNA. All primers were designed by the NCBI primer design tool (primer-BLAST) to have similar annealing temperature (optimal melting temperature was set to 60°C). Efficiency of all of the primers was assessed using the standard curve method using serial dilutions of the cDNA and plasmid target. The location of the IQSEC2 primers using NM_001114664 were between nucleotides 3139 and 3261 (spanning exons 9-11) representing sequences shared by the wild type, conditional and knockin S1474Q alleles.

Gene	Primer name	Description	Species	Forward /Reverse	Sequence
IQSEC2	Endo-IQSEC2	Primers for measuring mouse IQSEC2 mRNA	Mus musculus	Forward	GCAAGAAACCGGTCCTGTCG
				Reverse	AAATTTTGGTGACCTCCCGC

PGK1		Housekeeping gene	Mus musculus	Forward	CACCGAGCCCATAGCTCCAT
				Reverse	CTGCAACTTTAGCGCCTCCC

Table S3. Proteins with similar ligand binding site as the C terminus of S1474Qfs*133

Rank	Cscore^{LB}	PDB Hit	TM-score	RMSD^{a}	IDEN^{a}	Cov.	BS-score	Lig. Name	Download Complex	Predicted binding site residues
1	0.01	2wssL	0.455	5.28	0.053	0.854	0.12	ANP	complex1.pdb.gz	12,13,15
2	0.01	2wpdA	0.446	5.17	0.092	0.847	0.21	ATP	complex2.pdb.gz	12,17,110
3	0.01	2wssC	0.449	5.21	0.053	0.854	0.23	ANP	complex3.pdb.gz	10,13,14
4	0.01	2wojA	0.398	5.14	0.048	0.737	0.12	UUU	complex4.pdb.gz	102,103,104,125
5	0.01	1e79B	0.451	5.18	0.053	0.839	0.15	ADP	complex5.pdb.gz	13,14,17,18
6	0.01	1efrA	0.267	5.52	0.031	0.518	0.22	III	complex6.pdb.gz	10,11,12,13,14

(a) Cscore^{LB} is the confidence score of predicted binding site. Cscore^{LB} values range in between [0-1]; where a higher score indicates a more reliable ligand-binding site prediction.

(b) BS-score is a measure of local similarity (sequence & structure) between template binding site and predicted binding site in the query structure. Based on large scale benchmarking analysis, we have observed that a BS-score >1 reflects a significant local match between the predicted and template binding site.

(c) TM-score is a measure of global structural similarity between query and template protein.

(d) RMSD^{a} the RMSD between residues that are structurally aligned by TM-align.

(e) IDEN^{a} is the percentage sequence identity in the structurally aligned region.

(f) Cov. represents the coverage of global structural alignment and is equal to the number of structurally aligned residues divided by length of the query protein.
